# Alcohol marketing versus public health: David and Goliath?

**DOI:** 10.1186/s12992-021-00696-2

**Published:** 2021-04-12

**Authors:** Mary Madden, Jim McCambridge

**Affiliations:** Department of Health Sciences, University of York, Seebohm Rowntree Building, Heslington, York, YO10 5DD UK

**Keywords:** Commercial determinants of health, Alcohol, Public health, Alcohol marketing, Alcohol industry, Alcohol policy, Global health

## Abstract

**Background:**

Alcohol harms are rising globally, and alcohol policies, where they exist, are weak or under-developed. Limited progress has been made since the formulation of the World Health Organisation (WHO) Global Strategy in 2010. WHO is seeking to accelerate progress in implementing international efforts to reduce the harmful use of alcohol. The threat to global health posed by tobacco is well understood by policy communities and populations globally; by contrast alcohol is much less so, despite available evidence.

**The competition for epistemic authority:**

Global alcohol corporations have sought to become trusted sources of advice for policy makers and consumers, while continuing to grow their markets. Evidence-informed public health messaging faces formidable competition from transnational corporations as the worlds of corporate and political communications, social and mainstream media become increasingly linked, presenting new opportunities for corporate actors to shape global health governance. Alcohol messaging that uses means of persuasion tied to industry agendas does not tell a clear story about commercial determinants of health, and does not contribute to health improvement. On the contrary, the basic tenets of an evidence-informed population-based approach are denied and the policy measures supported by high quality evidence are being opposed, because they are inimical to commercial interests. A David and Goliath metaphor for this state of affairs, which seems to fit at first glance, may unwittingly reinforce the status quo.

**Conclusion:**

Public opinion on alcohol and policy issues varies across time and place and can be influenced by dedicated public health interventions. Alcohol marketing dominates people’s thinking about alcohol because we currently allow this to happen. Greater ambition is needed in developing countermarketing and other interventions to promote evidence-informed ideas with the public. Alcohol policies need to be further developed, and implemented more widely, in order to arrest the growing burden of alcohol harms across the world.

## Background

Alcohol policies, where they exist, are usually weak or under-developed in the face of the challenges with which they contend [1]. The current global annual death toll of 3 million is forecast to rise, particularly in low and middle income countries [2]. A minority of the world’s population drinks alcohol, so there is a large market to be developed [1]. A small number of corporations now produce most of the beer and spirits consumed across the world, creating an oligopoly [3]. Building consumer relationships with distinctive brands, creatively tailored to appeal to targeted audiences, helps provide competitive edge for individual companies [4]. This also conveys a sense of proliferating choice, even when innovations in production are absent. The worlds of corporate and political communications, social and mainstream media have become increasingly linked, presenting new opportunities for corporate actors to shape global health governance [5]. Extensive resources are deployed to exploit these opportunities and close relationships are built with key political actors through lobbying [6]. It is a marketing truism that those who set the frame control the agenda [7].

Evidence-informed public health messaging thus faces formidable competition from transnational alcohol corporations. This resembles in part a contest over epistemic authority, as corporations seek to become trusted sources of advice for policy makers and consumers, while continuing to grow their markets [8]. There is a growing mismatch between the expansion of global markets and efforts at national regulation [9]. A World Health Organisation (WHO) Action plan (2022–2030) is now proposed to accelerate progress in implementing international efforts to reduce the harmful use of alcohol [10]. This is needed because of the limited progress made since the formulation of a Global Strategy in 2010 [11, 12]. It can sometimes seem to public health interests that they are David, daring to hope to win against the odds, and the corporation is Goliath. Given that the actions that need to be taken are largely well established in the alcohol policy evidence, David should be winning by now. To get off the back foot, we need to further develop our understanding of the changing nature of the challenge.

## The competition for epistemic authority

### No level playing field

Corporate communication campaigns are now not only about introducing and seeking views on particular products and brands, but leading opinion more broadly in market friendly directions [13]. Corporate communicators take a strategic approach to the identification and segmentation of publics, cultivating relationships to influence consumer and public opinion and promoting reasons to care about brands and industry interests [14]. Because of the harmful nature of their products, alcohol producers are deeply invested in branding themselves as good corporate citizens [15, 16]. Corporate social responsibility initiatives like Drinkaware in the UK work as a form of implicit alcohol industry branding, managing conflicts between corporate and public health interests [17–20]. Such initiatives are produced within a corporate market logic focused on sustaining profit growth which indirectly promotes product consumption as responsible and normal [19, 21].

As well as publishing misinformation for the public [22, 23], the alcohol industry also funds and publishes research which casts doubt on scientific evidence about product harms and policy responses, some of which is designed to emphasise purported benefits [24–26]. The basic tenets of an evidence-informed population-based approach are denied and the policy measures supported by high quality evidence are opposed, because they are inimical to commercial interests [27, 28]. Extensive political lobbying and stakeholder marketing has created key networks and partnerships in many countries and, together with free market think tanks, provided persuasive rationales to secure preferred policy directions [8, 29]. The power imbalance raises important market ethics issues [30], as well as obvious public health policy issues.

Corporate investment in shaping drinking norms and distracting from evidence on alcohol harms amounts to the cultivation of ignorance, as pioneered by the tobacco industry [31, 32]. The tobacco playbook has been adopted by other industries [33], and there are deep historical links between alcohol and tobacco companies [34, 35]. There is recent evidence of tobacco and alcohol collaborative efforts to undercut the credibility of science [36] and they have long sought to influence policy together [37]. This helps to explain why alcohol has been appositely described as a global health blind spot [38].

### Alcohol exceptionalism

The threat to global health posed by tobacco is well understood; alcohol much less so, despite a substantial epidemiological evidence base identifying it as a major contributor to the global burden of disease, disability and death [39]. The alcohol and tobacco industries depend on addiction and other harmful forms of consumption in their operating models, and make products that harm others as well as the individual consumer [40, 41]. ‘Tobacco exceptionalism’ refers to the ways we think of the tobacco industry and its products as uniquely dangerous, and in need of a unique model of governance [42]. Thinking about the alcohol industry in such ‘exceptional’ terms seems harder to grasp. The promotion of alcohol is widespread, yet the industry that produces it appears invisible, with product retail largely undertaken by other parties such as the ‘hospitality industry’ and supermarkets. The alcohol and tobacco industries are both responsible for non-communicable diseases [43], with alcohol also implicated in infectious diseases [44]. Both products cause multiple health harms including cancers, cardiovascular diseases and foetal damage. Alcohol additionally causes overdose, intoxication, violence, suicide, accidents, job loss, sexually transmitted infections, unintended pregnancy and family breakdown. As with smoking, many of the health harming impacts of drinking are cumulative, manifesting over the longer term, and like COVID-19, its impacts fall heaviest in socioeconomically disadvantaged communities [11]. Alcohol can also kill quickly [45].

A long tradition of drinking in some societies may account for an implicit acceptance, and the normalisation of alcohol harms, which may lead to a view that change is not possible. There is limited research on people’s perceptions of their own drinking and most of this is conducted with young people [46]. In such studies, as with the reinforcing effects of corporate social responsibility organisation messaging, personal risk is perceived as low [47]. A particular stereotypical and stigmatising view of the ‘alcoholic’ as ‘other’ serves to distance adult drinkers from recognition of risk or harm, regardless of their current health status or the quantity of alcohol actually consumed [48].

Reducing alcohol health harms inevitably means reducing the amount of the drug ethanol consumed, as this is the source of the harm [49]. Just as with tobacco, the most effective and cost-effective interventions are increasing the price of alcohol and reducing its physical availability and marketing [50]. Yet, alcohol remains a privileged, protected, and indeed ubiquitous product. The contrast with tobacco is increasingly stark. Alcohol has been defined as an ‘essential’ commodity in the UK and Australia during the COVID-19 pandemic, with off-sales protected and pubs the first places to open up as lockdowns eased [51–53]. Alcohol industry actors are exceptionally effective at distracting attention from responsibility for harm, the extent of policy interference, and the similarities with tobacco.

### Taking alcohol messaging upstream

There is gross asymmetry in the resources available in the research and public health arenas to produce, test and distribute high quality, well targeted, public-focused, messaging tailored for a range of media. It does look like something of a David and Goliath contest to oppose the interests of major transnational corporations and to intervene in an enjoyable well established social practice for many. Responsibility for the choice to drink rests with the individual, and somewhat less attention gets paid to the nature of the market, the information conditions under which that choice is exercised, and other constraints on individual choice. There are other asymmetries in play. The costs are borne by society, with the risks operating at individual, family, community and population levels, whilst corporations and their shareholders enjoy the benefits. Not drinking, or drinking less alcohol, can feel like opting out of an activity considered by others as central to relaxing and having fun.

The impoverished nature of public discourse on alcohol harms, means this is not an area where the public are currently clamouring to see more intervention. Marketing thus proceeds to lock in branded thinking about alcohol, in a vicious circle. Public opinion on alcohol and policy issues varies across time and place and can be influenced by public health interventions [54]. Existing evidence on mass media interventions is weak, and these are under-developed [55]. The ambition needed must match the sophistication of alcohol marketing in order to counter it. More needs to be done to understand how to get evidence-informed ideas out to the public and to create demand for population health measures.

Our work has identified the caution, scepticism and confusion with which ordinary drinkers receive advice from health professionals and the discomfort that health professionals experience in discussing drinking [56–58]. Even for those convinced that their drinking is not a ‘problem,’ being asked about alcohol use in a health context can elicit negative emotions, including feeling judged or guilty, making open conversation difficult. In such circumstances a brief chat will rarely be any match for the huge corporate investment in encouraging drinking, and the wider and long running shaping of how we think about alcohol.

Corporate messaging has long experimented with active persuasion; shaping preferences to match values or solve problems. Messengers use a range of strategies to obtain favourable policy environments, build constituencies, sow doubt, creatively pursue “defactualization” taking advantage of what the audience wants to hear, with a careful eye to making this all credible and coherent [59]. Opportunities for attracting attention and practicing persuasion have expanded with the rise of digital social media, giving rise to formidable persuasion industries [60]. Some countries provide financial incentives for alcohol industry marketing through their taxation policies. For example, in the US, in 2017 the top 10 alcohol producers were exempted from paying taxes on US $1.5 billion for beer advertising alone [61]. Marketing to consumers is far from only being about persuading people to make purchase decisions, but analysing customers’ choices and behaviours so they are not even aware their buying decisions are being scrutinised and influenced [62]. Data analytics underpin what has been understood as surveillance capitalism [63, 64]. Social media offers publishing platforms funded by data-driven advertising which are free of the regulations to which other forms of media are subject, with no responsibility for accuracy of content.

Alcohol messaging must contend with how alcohol and alcohol harm can be reframed to evoke a different way of thinking about the personal and policy choices for health to be improved. This requires countermarketing that addresses ideas that products are new, aspirational, and identity, socially or even health enhancing. Learning from the tobacco experience makes many lessons available on how to make progress in improving population health [38, 65]. The nature of the threat to global health means that the situation will get worse unless we embrace the many challenges posed by alcohol marketing.

Alcohol research has historically been predominantly focused on particular populations and forms of problematized drinking that have stereotypical and highly stigmatised and stigmatising foundations [66, 67]. Alcohol science has been built around the fundamentally flawed concept of alcoholism and the associated treatment movement, in part due to industry involvement since the 1940s [68]. Similarly, alcohol health messaging is currently framed in an extreme close up on the drinker and whether or not they are consuming responsibly [69, 70]. The appearance of placing limits on individual choices is seized on by industry messaging, which additionally stereotypes the meddling, moralising ‘expert’ and nanny telling people what to do. We could begin to develop the science of alcohol messaging simply by introducing a wider angle which includes the corporate context and the drug ethanol as characters within the messaging.

## Conclusion

Gerard Hastings wrote a key textbook on social marketing subtitled “why should the devil get all the best tunes?” [71] This rhetorical question invites us to invest more substantially in countermarketing ideas in improving health. Corporate actors, particularly in controversial sectors which damage health or the environment, are adept at myth making [72]. The alcohol companies, and the neoliberal fictions they contribute to and benefit from, position the state as Goliath, and the individual consumer as David. They use sophisticated tools of persuasion to ally themselves with David, producing a dystopian version of individual freedom which allocates responsibility for risk to individuals and renders invisible the processes of maximising shareholder wealth.

So many of the contemporary challenges in public health revolve around such contests with powerful global corporations, and alcohol is no different. The David and Goliath story has undergone many revisions in its re-telling to become a powerful metaphor for the potential of the plucky underdog. This metaphor is unhelpful if it keeps public health David on the back foot and stuck at the start of the contest. Alcohol marketing dominates people’s thinking about alcohol because we currently allow this to happen. We give corporations a license to operate, and we should look at the terms of the license, and revise them, to better protect public health. Some countries have complete bans on alcohol marketing and WHO recommends that such bans should be enforced where they exist, and comprehensive restrictions on advertising, sponsorship and promotion introduced where they do not. If that idea makes you uncomfortable, you might ask yourself why.

## Data Availability

Not applicable.
